# Factors Affecting Discharge to Home of Medical Patients Treated in an Intensive Care Unit

**DOI:** 10.3390/ijerph16224324

**Published:** 2019-11-06

**Authors:** Takayuki Shimogai, Kazuhiro P. Izawa, Minoru Kawada, Akira Kuriyama

**Affiliations:** 1 Department of Public Health, Graduate School of Health Sciences, Kobe University, Kobe 654-0142, Japan; tak813hir@gmail.com; 2 Department of Rehabilitation, Kobe City Medical Center General Hospital, Kobe 650-0047, Japan; 3 Cardiovascular Stroke Renal Project (CRP), Kobe 654-0142, Japan; 4 Department of Rehabilitation, Kurashiki Central Hospital, Kurashiki 710-8602, Japan; mkawada0805@gmail.com; 5 Emergency and Critical Care Center, Kurashiki Central Hospital, Kurashiki 710-8602, Japan

**Keywords:** intensive care unit, medical patients, discharge, rehabilitation, early mobilization, physical therapy

## Abstract

The purpose of this study was to examine the factors affecting the discharge to home of medical patients treated in an intensive care unit, including elements of in-hospital rehabilitation and prehospital movement ability. The participants of this retrospective cohort study were medical patients treated in an intensive care unit (ICU) and who began rehabilitation in ICU. We assessed the participants in the ICU and analyzed data on patient background, hospitalization, and rehabilitation status. There were 155 ICU patients available for analysis. A multivariable logistic regression model identified the four variables of age (OR 1.06, 95% CI 1.02–1.09), APACHE II score (OR 1.12, 95% CI 1.04–1.24), independence in home life before admission (OR 7.10, 95% CI 1.65–30.44), and standing within 5 days of admission (OR 6.58, 95% CI 2.60–16.61) as factors significantly related to discharge from hospital to home. Independence of home life before admission and early start of standing were identified as factors strongly related to discharge to home. The degree of independence in living before hospital admission and progress toward early mobilization are helpful when considering an ICU patient’s discharge destination.

## 1. Introduction

In the critical care area, intensive care management has improved over the last decade. As a result, the survival rate of critically ill patients has improved [1,2], and the outcome after discharge following treatment in an intensive care unit (ICU) can vary. For example, some ICU patients recover within a short time and are discharged to home (defined as being discharged from the hospital directly to home without transfer to another facility such as an intermediate care facility or nursing home), others cannot fully recover their function after discharge, some require long-term hospitalization and further care after hospital discharge, and some patients enter nursing homes [3,4].

Also, the rate of direct-to-home discharges from the ICU also tend to increase [5] because the beds in the ICU and on wards are becoming unavailable and the support of local communities and the quality of medical care have improved [6].

In fact, it is reported that about 25% to 45% of patients treated in the ICU were not discharged home [3,7]. In addition, 31.6% of such patients are rehospitalized within 30 days, and the rate of unplanned readmission is reported to be 23.2% [8].

If ICU patients can be discharged to the next appropriate destination, their duration of hospitalization can be shortened, which will reduce iatrogenic morbidity and lead to cost reductions and effective use of medical resources [5]. With the diversification of sites to which patients can be discharged and to determine the correct discharge destination, it is important to decide which ICU patients can be transferred to a care facility and or safely return home. For this purpose, it is necessary to evaluate the risk factors related to home discharge during hospitalization.

In this current situation, some reports have examined the risk factors of discharge outcome. Harrison et al. suggested dementia and elderly women with high dependence as predictors of discharge to a long-term care facility after acute admission [9]. As well, mechanically-ventilated patients and those with severe cognitive dysfunction or poor physical function and/or mobility difficulties are also at risk for discharge to a nursing home [7].

Rehabilitation strategies for severely-ill emergency patients are said to promote the restoration of previous activity with respect to muscle strength and ability [10]. One of the effects of early rehabilitation in the ICU also relates to early improvement of the activities of daily living (ADL) [11,12]. In addition, early mobilization is reported to improve the rate of discharge to home of patients who were treated with mechanical ventilation in the ICU [13].

Therefore, factors related to the state of rehabilitation in ICU patients should be taken into consideration when planning discharge. Nevertheless, in relation to these previously mentioned factors [6,7,9,13], no studies of factors related to discharge have considered the effects of early rehabilitation in the ICU during hospitalization.

Thus, we hypothesized that early rehabilitation might be associated with discharge to home of ICU patients. Based on this hypothesis, the purpose of this study was to examine the factors affecting discharge to home of medical patients in the ICU.

## 2. Materials and Methods 

### 2.1. Study Design and Patients

This was a retrospective cohort study conducted from April 2013 to June 2015 in the Emergency and Critical Care Center of Kurashiki Central Hospital, Kurashiki, Japan.

Consecutive participants were recruited between April 2013 and June 2015. Participants included medical patients who were treated in and also started rehabilitation in the ICU. Patients were excluded from the study for the following reasons: transfer to a general ward with no rehabilitation occurring in the ICU, death during hospitalization, refusal of rehabilitation.

### 2.2. Study Procedures

We collected data on patient background, hospitalization, and rehabilitation status.

Patient background included disease (classified by the International Statistical Classification of Diseases and Related Health Problems 10th Revision (ICD-10)), age, sex, body mass index, SOFA (Sequential Organ Failure Assessment) score [14], APACHE II (Acute Physiology and Chronic Health Evaluation II) score [15], criteria for evaluating the degree of independence (degree of “bed riddenness”) of the disabled elderly patients in performing ADL before admission, and the rate of living alone.

Variables during hospitalization included serum albumin (Alb) value at the times of admission and discharge, peak value of C-reactive protein during hospitalization (peak CRP), peak white blood cell count while hospitalized (peak WBC), use of cardiotonic drugs, Medical Research Council (MRC) score at the beginning and end of rehabilitation at discharge, diagnosis of delirium, treatment with mechanical ventilation, and length of hospital stay in days.

Status of rehabilitation included the number of days from admission to the start of rehabilitation, the first time the patient sat at the edge of the bed, start of standing within 5 days of admission, and the Functional Independence Measure score (FIM; exercise items and cognitive items) [16] at the start and end of rehabilitation.

### 2.3. Criteria for Evaluation of ADL Before Admission

“Criteria for evaluating the degree of independence (degree of ‘bed riddenness’) of disabled elderly persons in performing activities of daily living (in Japanese)” are criteria proposed by the Ministry of Health, Labor and Welfare of Japan, and although not internationally standardized [17,18], they are widely used in Japan [19]. In this standard, subjects classified as rank J have some degree of disability, but they are almost completely independent in daily activities and can go out without assistance. Subjects classified as Rank A are almost independent in everyday life activities but cannot go out without assistance. Subjects classified as Rank B require a certain level of assistance to carry out indoor activities and are mainly confined to bed day and night, but they can sit on the bed. Subjects classified as Rank C are bed-ridden and require assistance for toileting, eating, and changing of clothes [18].

In the present study, we used these as the criteria of ADL before admission. In addition, we defined the patients of ranks J and A as having independence in home life and the patients of ranks B and C as being bedridden and divided the patients according to these two pre-hospital ADL abilities.

### 2.4. Assessment of Muscle Strength

After the patient awoke on the morning following the first day of admission or regained consciousness if unconscious, muscle strength was measured in all four limbs using the MRC scale, which uses values ranging from 0 (tetraplegia) to 60 (normal muscle strength) [20]. This scale is a graded summation of the strength of 6 muscle groups tested bilaterally. These muscle groups are the arm abductors, forearm flexors, wrist extensors, leg flexors, knee extensors, and dorsal foot flexors. The MRC scale score is calculated by recording the muscle strength scores according to 5 grades of power for the 6 muscle groups, each on the left and right, and summing the scores [20].

### 2.5. Assessment of Disability

The ability to sit on the edge of the bed is referred to as sitting ability of Hoffer’s classification [21], which represents that the patient sits on the edge of the bed with his/her feet on the floor and releases his/her hands from the bed. The patient is then evaluated according to three classifications: maintains a stable sitting position (independent sitter), holds a sitting position with support of the hands (hands-dependent sitter), or impossible to sit (propped sitter). In this study, when sitting on the edge of the bed was performed for the first time, maintenance of a sitting position was defined if the patient was either an independent sitter or hands-dependent sitter.

We considered “standing up” as the point at which a patient’s activity greatly increases for rehabilitation, and thus we included standing within 5 days of admission in the measurement items. In Europe and the United States, early mobilization is considered to be physical activity that takes place within 2 to 5 days of admission [22,23]. Therefore, we defined patients who could stand within 5 days of admission as getting up early.

### 2.6. Ethical Considerations

This study is in accordance with the Declaration of Helsinki. The Kurashiki Central Hospital Institutional Review Committee on Human Research approved this study (approval no. 1898). This study omitted patient consent because of a retrospective study.

### 2.7. Statistical Analysis

We classified the patients who were discharged from our hospital to home into the home discharge group and those who were transferred from our hospital to another care facility into the transfer group. Univariate analysis was used to compare each item of the patient’s background, variables during hospitalization, and rehabilitation status between the home discharge group and the transfer group. Comparisons were performed using a Mann-Whitney U test for continuous variables, and chi-square test for binary variables. A two-tailed p-value <0.05 was considered to indicate statistical significance.

Multivariable logistic regression models were used to examine the effect of the variables on home discharge of the ICU patients. Selection of variables was based on previous literature [5,6]. Patients discharged directly to home from the ICU have been found to be young, healthy, and to have fewer comorbidities than those not discharged directly to home [5,6]. For this reason, we added age and the APACHE II score as variables in this multivariate analysis. The final multivariable model was developed by including the variables whose statistical significance was p ≤ 0.05, and the variables considered to be multi-collinear were excluded. We simultaneously entered variables that we considered the risk factors of discharge to home of the patients in the multivariate logistic regression. Statistical analysis was performed by IBM SPSS 24.0 J statistical software (IBM SPSS Japan, Inc., Tokyo, Japan). 

## 3. Results

**Figure 1:**
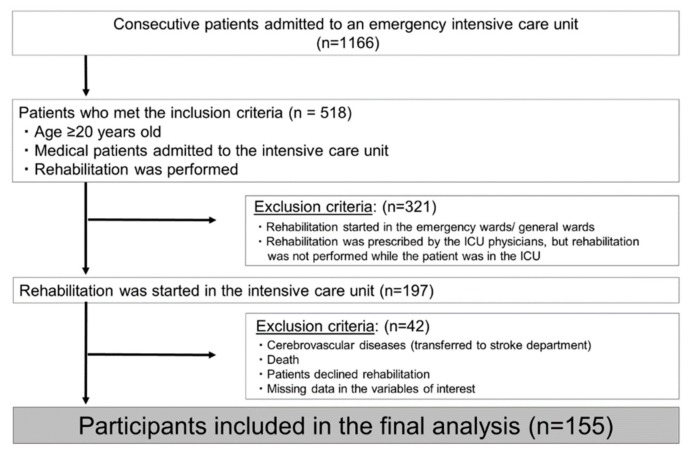
Flow of patient selection.

From April 2013 to July 2015, 1166 consecutive patients were admitted to our emergency medical center in Kurashiki Central Hospital. Among these, the ICU physicians requested rehabilitation for 518 patients. Among them, 197 patients were included the rehabilitation cases started in ICU. 42 patients (with cerebrovascular disease who were transferred to the stroke unit, death, denied rehabilitation and missing data for the evaluation items) were excluded. As a result, 155 patients were included in the final analysis (Figure 1). About two-thirds (n = 95, 61.3%) of these patients were discharged home directly from our hospital.

Patient characteristics of the study cohort are shown in Table 1. As a result of classification based on the ICD-10, the distribution of patients mostly comprised those with sepsis and respiratory diseases, followed by digestive system diseases.

Among patient background variables, there were significant differences between the two groups in terms of age, SOFA, APACHE II score, and ADL before hospitalization. Among the hospitalization variables, significant differences between the two groups were confirmed for Alb at hospitalization and MRC score at the start of rehabilitation (Table 2).

Among the variables on rehabilitation between the two groups, significant differences were present for the start date of standing within 5 days of admission, length of hospital stay, and FIM (motor/cognitive) at the start of rehabilitation.

The results of the multivariable analysis are reported in Table 3. The multivariable logistic regression model showed the following four variables to be significantly associated with an increased rate of home discharge: age (OR 1.06, 95% CI 1.02–1.09), APACHE II score (OR 1.12, 95% CI 1.04–1.24), independence in home life before admission (OR 7.10, 95% CI 1.65–30.44), and standing within 5 days of admission (OR 6.58, 95% CI 2.60–16.61). The model showed good calibration (Hosmer-Lemeshow Goodness-of-Fit test: p = 0.63, percentage of correct classifications: 79.4%).

## 4. Discussion

The present study suggested that age, independence in home life before admission, APACHE II score, standing within 5 days of admission, and admission Alb value were the independent factors related to discharge to home of medical patients admitted to an ICU. In particular, independence in home life before admission and standing within 5 days of admission were the strongest factors affecting discharge to home.

### 4.1. Independence at Home Before Admission

In ICU patients or general medical patients, the important variable that predicts hospital outcome is reported to be the baseline level of activity at admission [24,25]. Furthermore, another previous study suggests that pre-ICU frailty is associated with increased post-ICU disability and the rate of new nursing home admissions [26]. From these facts, independence at home before admission is important as a predictive factor related to home discharge, and this was suggested from our research results.

### 4.2. Starting Date of Standing

Rehabilitation for ICU patients with critical illness is important to aid in their return to prehospital activity [10]. In particular, a recent meta-analysis concluded that early rehabilitation can increase the possibility of walking without assistance at hospital discharge [12]. Early rehabilitation in the ICU has been shown to improve the subsequent ADL of hospitalized patients [11,27]. It has also been reported that physical function leading to reacquisition of ADL can be improved by getting the patient out of bed early and engaging them in active exercise from an early stage [28,29]. It is important for patients to stand as soon as possible from the results of this study. Therefore, for ICU patients, we recommend conducting a program of early rehabilitation, including standing at the bedside, to help return ADL capabilities at discharge.

### 4.3. Factors Affecting Discharge to Home

Lower age and higher test scores (The Physical Function in Intensive Care Test (PFIT-s), Functional Status Score for the ICU (FSS-ICU), and ICU Mobility Scale (IMS)) at ICU discharge are significant factors in determining discharge to home [30]. Also, older ICU survivors with a low burden of comorbidities and little to no disability at hospital discharge have generally good outcomes [31,32]. The risk for discharge to a care facility was greatest for hospitalized patients characterized as having poor strength and/or mobility [7].

From these findings, early rehabilitation is important for achieving a good outcome as it is largely related to ADL independence before admission and mobility after hospitalization, and to age, disease, and complications. Thus, all of these factors need to be evaluated when determining the appropriateness of discharge to home of patients treated in the ICU. As indicated by this study, the patient’s degree of independence in living before admission to the hospital and the progress made toward early mobilization also help when considering the discharge destination.

### 4.4. Implications

To our knowledge, this is the first study to show that the progress of in-hospital rehabilitation is a relevant factor related to discharge to home of medical patients admitted to an ICU. ICU rehabilitation offers many benefits, including reduced length of ICU and hospital stays, increased number of ventilator-free days, and improved peripheral and respiratory muscle strength, physical function, and health-related quality of life (HRQOL) [11,12]. In addition to these existing reports, the results of this study may encourage rethinking of the implications of early rehabilitation and the consideration of interventions to reduce the need for discharge to places other than home. As the present study shows, it is clear that discharge to home is related to various factors, and further study should be undertaken in a prospectively to determine whether discharge outcomes improve when adjusting variables based on the results.

### 4.5. Limitations

There are some limitations in our present study. First, this study is for patients with medical diseases in ICU. We summarized the patient characteristics of the disease using ICD-10, and as a result, classified into many disease types. Although there were various types of diseases, we were unable to investigate the course of each individual disease because this study targets all medical diseases. Second, this study was a retrospective study, and we failed to investigate some potentially important factors might not have been investigated due to the lack of such data. For example, some test scores (PFIT-s, FSS-ICU, and IMS) and comorbidities are reportedly factors that determine home discharge when leaving the ICU [30,31,32]. However, this study could not investigate them during the hospitalization. Further, the participants in this study were relatively old. The presence of family members and caregivers, the home environment, and home care services may be important when clinicians consider discharging patients to home; however, we failed to investigate the impact of these factors due to the lack of such data. Prospective studies that examine whether these factors have an influence on the discharge destination of ICU patients. Third, this is a single-center study that was conducted in a representative city of Japan. Although it seems to reflect the general population in Japan, regional characteristics must be considered when planning patient discharge.

## 5. Conclusions

This We identified factors affecting discharge to home of medical patients treated in an ICU. In addition to generally reported factors such as age and illness severity, independence in the patients before admission home life and their ability to stand early after admission were identified. Therefore, the degree of independence in home life before admission to hospital and the progress of early mobilization should aid in considering the appropriate discharge destination of ICU patients.

## Figures and Tables

**Table 1:** Patient characteristics (classified according to the ICD-10*).

Major Classification	n	Minor Classification
I. Certain infectious and parasitic diseases	46	Sepsis 46
X. Diseases of the respiratory system	37	Chronic obstructive pulmonary disease (COPD)—acute exacerbation 4, interstitial pulmonary diseases 5, asthma 3, hemorrhage from other sites in respiratory passages 1, bacterial pneumonia 14, pneumonitis due to food and vomiting 1, drug-induced interstitial lung disorders, unspecified 2, drowning and nonfatal submersion 2, bronchus or lung, unspecified 1, acute respiratory failure 3, other and unspecified abnormalities of breathing (CO_2_ narcosis) 1
XI. Diseases of the digestive system	21	Gastrointestinal hemorrhage 12, acute pancreatitis 5, esophageal varices 2, gastric ulcer 1, peritonitis 1
XIX. Injury, poisoning and certain other consequences of external causes	18	Other and unspecified drugs/medicaments and biological substance (acute drug addiction) 13, hypothermia 2, injury of muscles and tendons of unspecified body region 1, injury of intercostal blood vessels 1, toxic effect of carbon monoxide 1
IV. Endocrine, nutritional and metabolic diseases	15	Elevated blood glucose level 3, hypoglycemia, unspecified 2, diabetes mellitus with ketoacidosis 4, other disorders of electrolyte and fluid balance, not elsewhere classified 4, hypothyroidism, unspecified 1, acidosis (due to alcohol) 1
VI. Diseases of the nervous system	7	Encephalitis, myelitis and encephalomyelitis 3, epilepsy 2, disorder of autonomic nervous system 1, myasthenia gravis 1
XVIII. Symptoms, signs and abnormal clinical and laboratory findings, not elsewhere classified	3	Coma, unspecified 1, unspecified adverse effect of drug or medicament (serotonin syndrome) 1, Hemoptysis 1
XIV. Diseases of the genitourinary system	5	Acute renal failure 5
IX. Diseases of the circulatory system	1	Cardiac arrest 1
XII. Diseases of the skin and subcutaneous tissue	1	Bullous erythema multiforme (Stevens-Johnson syndrome) 1
XIII. Diseases of the musculoskeletal system and connective tissue	1	Other specified disorders of muscle (rhabdomyolysis) 1

* International Statistical Classification of Diseases and Related Health Problems 10th Revision.

**Table 2:** Summary comparing home discharge and transfer.

	Home Discharge	Transfer	P-Value
	n = 95	n = 60	
Age	66.0 (51.0, 74.0)*	75.5 (65.8, 82.3) *	<0.001
Sex (male/female)	61/34	37/23	0.75
SOFA score	6.0(3.5, 9.0) *	7.0 (4.8, 9.3) *	0.049
APACHE II score	16.0 (11.5, 20.0) *	21.5 (15.0, 25.0) *	<0.001
Independence at home before admission (%)	95.8% (91/95)	76.7% (46/60)	<0.001
Peak CRP (mg/L)	10.32 (4.16, 17.14) *	14.88 (5.69, 19.74) *	0.26
Peak WBC (×10^3^/μL)	14.20(10.05, 18.90) *	15.25 (10.00, 21.47) *	0.39
Admission albumen (g/dL)	3.00 (2.60, 3.80) *	2.70 (2.20, 3.40) *	0.009
Use of cardiotonic drugs (%)	23.2% (22/95)	35.0% (21/60)	0.109
Mechanical ventilation (%)	74.7% (71/95)	73.3% (44/60)	0.85
Delirium (%)	9.5 (9/95)	16.7 (10/60)	0.184
Rehabilitation start date (day)	1.0 (1.0, 2.0) *	1.0 (1.0, 2.0) *	0.59
Initial MRC score	52 (48, 55) *	46 (37, 49.5) *	<0.001
Hands-dependent sitter (%)	89.5 (85/95)	51.7 (31/60)	<0.001
Standing within 5 days of admission (%)	84.2 (80/95)	51.7 (31/60)	<0.001
Motor FIM (start)	13.0 (13.0, 16.5) *	13.0 (13.0, 13.0) *	0.012
Cognitive FIM (start)	23.0 (10.5, 29.0) *	5.5 (5.0, 14.3) *	<0.001
Hospital stay (days)	18.0 (12.0, 29.0) *	29.5 (19.0, 49.8) *	<0.001

Legends: SOFA, Sepsis-related Organ Failure Assessment; APACHE II, Acute Physiology and Chronic Health Evaluation II; CRP, C-reactive protein; WBC, white blood cell; MRC, Medical Research Council; FIM, Functional Independence Measure. *Median (interquartile range (IQR)): Continuous data are presented as median and IQR. Categorical data (including sex and discharge diagnoses) are presented as total number and percentage in this study.

**Table 3:** Multivariate analysis of factors relating to home discharge or transfer*.

Variable	P-Value	OR	95% CI
Age	0.001	1.06	1.02 – 1.09
APACHE II score	0.002	1.12	1.04 – 1.20
Independence at home before admission	0.008	7.10	1.65 – 30.44
Standing within 5 days of admission	<0.001	6.58	2.60 – 16.61

OR, odds ratio; CI, confidence interval; APACHE, Acute Physiology and Chronic Health Evaluation. *Hosmer-Lemeshow test, p = 0.958. Percentage of correct classifications: 79.4%.
